# Supplementary material for: Identification of photoperiod-induced specific miRNAs in the adrenal glands of Sunite sheep (Ovis aries)
Source: Front Vet Sci. 2022 Jul 22;9:888207. doi: 10.3389/fvets.2022.888207 (PMC9354845; doi:10.3389/fvets.2022.888207)
Supplement: Supplementary file 1 [file Data_Sheet_1.ZIP › Supplementary file/miRDeep2 file.zip!/miRDeep2 file/Know_Structure/SPLP42A3/oar-mir-374a.pdf]

miRBase precursor	: oar-mir-374a
Total read count	: 21069
oar-miR-374a read count	: 18119
remaining reads	: 2950

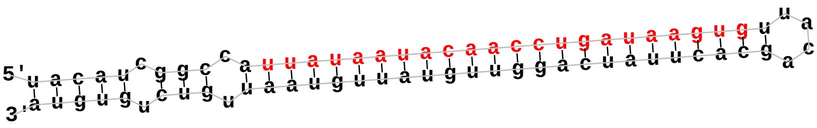

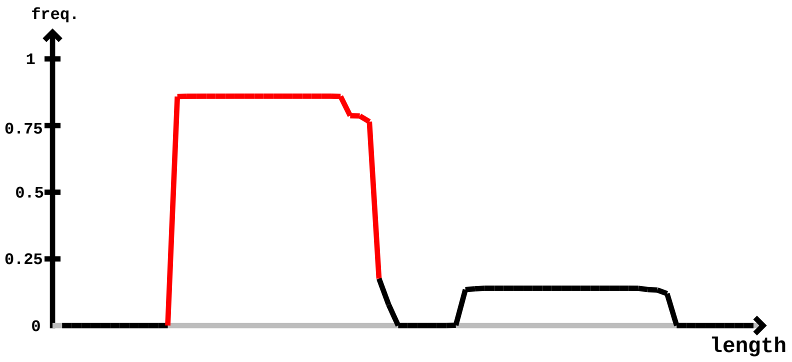


```
                oar-miR-374a
5'- uacaucggccauuauaauacaaccugauaaguguuacagcacuuaucagguuguauuguaauugucugugua   -3'    exp
    ((((((.(((.((((((((((((((((((((((((....)))))))))))))))))))))))).))).)))))   reads     mm         sample
    ..........auGauaauacaaccugauaagu........................................   1         1          seq
    ..........auuauaauacaaccugauaagu........................................   1         0          seq
    ..........auuauaauacaaccugauaagug.......................................   1         0          seq
    ...........Cuauaauacaaccugau............................................   1         1          seq
    ...........uuCuaauacaaccugau............................................   1         1          seq
    ...........Auauaauacaaccugau............................................   1         1          seq
    ...........uuauaauacaaccugau............................................   18        0          seq
    ...........uuauaauacaaccugaua...........................................   1430      0          seq
    ...........Cuauaauacaaccugaua...........................................   16        1          seq
    ...........uCauaauacaaccugaua...........................................   4         1          seq
    ...........uuauaauacaacAugaua...........................................   1         1          seq
    ...........uuaAaauacaaccugaua...........................................   22        1          seq
    ...........uuauUauacaaccugaua...........................................   4         1          seq
    ...........uuaCaauacaaccugaua...........................................   28        1          seq
    ...........uuauaauacaaccugauU...........................................   12        1          seq
    ...........uuauaauacaaccugGua...........................................   3         1          seq
    ...........uuauaauacaaAcugaua...........................................   1         1          seq
    ...........uuauaauacaaccugauaa..........................................   19        0          seq
    ...........uuauaauacaaccugauaag.........................................   442       0          seq
    ...........uGauaauacaaccugauaag.........................................   1         1          seq
    ...........uuaCaauacaaccugauaag.........................................   7         1          seq
    ...........uuauaauacaaccugGuaag.........................................   2         1          seq
    ...........uuauaauacaaccugauaagu........................................   12239     0          seq
    ...........uGauaauacaaccugauaagu........................................   4         1          seq
    ...........uuaCaauacaaccugauaagu........................................   140       1          seq
    ...........uuaCaauacaaccugauaagug.......................................   17        1          seq
    ...........uuauaauacaaccugauaagug.......................................   2026      0          seq
    ...........uuauaauacaaccugauaagugu......................................   1648      0          seq
    ...........uuauaauacaaccugauaaguguuacagc................................   1         0          seq
    ............uauaauacaaccugauaagu........................................   12        0          seq
    ............uauaauacaaccugauaagug.......................................   5         0          seq
    ............uauaauacaaccugauaagugu......................................   7         0          seq
    .............auaauacaaccugauaagu........................................   1         0          seq
    .............auaauacaaccugauaagug.......................................   1         0          seq
    ..............uaauacaaccugauaagugu......................................   1         0          seq
```

oar-miR-374a

```
uacaucggccauuauaauacaaccugauaaguguuacagcacuuaucagguuguauuguaauugucugugua
..............uaauacaaccugauaagugC......................................    1       1       seq
..............uaauacaaccugauaaguguuacagcacu.............................    1       0       seq
.................uacaaccugauaagugu......................................    1       0       seq
........................................acuuaucagguuguauugu.............    1       0       seq
........................................acuuaucagguuguauugua............    2       0       seq
........................................acuuaucagguuguauuguaa...........    2       0       seq
........................................acuuaucagguuguauuguaau..........    5       0       seq
........................................acuuaucagguuguauuguaauu.........    11      0       seq
.........................................cuuaucagguuguauug..............    1       0       seq
.........................................cuuaucagguuguauugu.............    3       0       seq
.........................................cuuaucagguuguauugua............    92      0       seq
.........................................cuuCucagguuguauugua............    3       1       seq
.........................................cuuaucagguuguauuAua............    2       1       seq
.........................................cuuaucagguuguauuguaa...........    37      0       seq
.........................................cuuaucagguuguauuguaau..........    268     0       seq
.........................................cuuaucagguuAuauuguaauu.........    2       1       seq
.........................................cuuaucagguuguauuguaauu.........    2408    0       seq
..........................................uuaucagguuguauuguaau..........    6       0       seq
..........................................uuaucagguuguauuguaauu.........    62      0       seq
...........................................uaucagguuguauuguaau..........    1       0       seq
...........................................uaucagguuguauuguaauu.........    39      0       seq
...........................................uaucagguuguauuguaauug........    1       0       seq
...........................................uaucagguuguauuguaauugu.......    2       0       seq
```
